# Increased expression of the HDAC9 gene is associated with antiestrogen resistance of breast cancers

**DOI:** 10.1002/1878-0261.12505

**Published:** 2019-06-12

**Authors:** Aurélien Linares, Said Assou, Marion Lapierre, Erwan Thouennon, Céline Duraffourd, Carole Fromaget, Abdelhay Boulahtouf, Gao Tian, Jiafu Ji, Ozgur Sahin, Eric Badia, Nathalie Boulle, Vincent Cavaillès

**Affiliations:** ^1^ IRCM, Institut de Recherche en Cancérologie de Montpellier France; ^2^ INSERM, U1194 Montpellier France; ^3^ Université Montpellier France; ^4^ ICM Montpellier France; ^5^ IRMB, Institute for Regenerative Medicine & Biotherapy Montpellier France; ^6^ INSERM, U1183 Montpellier France; ^7^ Laboratoire de Biopathologie des Tumeurs CHU Arnaud de Villeneuve Montpellier France; ^8^ Key Laboratory of Carcinogenesis and Translational Research Ministry of Education Department of Gastrointestinal Surgery Peking University Cancer Hospital & Institute Beijing China; ^9^ Department of Drug Discovery and Biomedical Sciences University of South Carolina Columbia SC USA

**Keywords:** antiestrogen resistance, breast cancer, cell proliferation, estrogen receptor, histone deacetylase

## Abstract

Estrogens play a pivotal role in breast cancer etiology, and endocrine therapy remains the main first line treatment for estrogen receptor‐alpha (ERα)‐positive breast cancer. ER are transcription factors whose activity is finely regulated by various regulatory complexes, including histone deacetylases (HDACs). Here, we investigated the role of HDAC9 in ERα signaling and response to antiestrogens in breast cancer cells. Various Michigan Cancer Foundation‐7 (MCF7) breast cancer cell lines that overexpress class IIa HDAC9 or that are resistant to the partial antiestrogen 4‐hydroxy‐tamoxifen (OHTam) were used to study phenotypic changes in response to ER ligands by using transcriptomic and gene set enrichment analyses. Kaplan–Meier survival analyses were performed using public transcriptomic datasets from human breast cancer biopsies. In MCF7 breast cancer cells, HDAC9 decreased ERα mRNA and protein expression and inhibited its transcriptional activity. Conversely, HDAC9 mRNA was strongly overexpressed in OHTam‐resistant MCF7 cells and in ERα‐negative breast tumor cell lines. Moreover, HDAC9‐overexpressing cells were less sensitive to OHTam antiproliferative effects compared with parental MCF7 cells. Several genes (including MUC1, SMC3 and S100P) were similarly deregulated in OHTam‐resistant and in HDAC9‐overexpressing MCF7 cells. Finally, HDAC9 expression was positively associated with genes upregulated in endocrine therapy‐resistant breast cancers and high HDAC9 levels were associated with worse prognosis in patients treated with OHTam. These results demonstrate the complex interactions of class IIa HDAC9 with ERα signaling in breast cancer cells and its effect on the response to hormone therapy.

**Abbreviations**
CGH: comparative genomic hybridization
ERE: estrogen‐responsive element
ER: estrogen receptor
ESR1: estrogen receptor 1
GO: Gene Ontology
GSEA: gene set enrichment analysis
HDAC: histone deacetylase
HDI: HDAC inhibitor
IPA: Ingenuity Pathway Analysis
KO: knockout
MCF7: Michigan Cancer Foundation‐7
MTT: 3‐(4,5‐dimethylthiazol‐2‐yl)‐2,5‐diphenyltetrazolium bromide
NRIP1: nuclear receptor interacting protein 1
OHTam: 4‐OH‐tamoxifen
OHTR: OHTam‐resistant
PR: progesterone receptor
SD: standard deviation
TBP: TATA‐binding protein

## 1 Introduction

Estrogens are important regulators of gene expression in target tissues, such as the mammary gland, and their mitogenic action has a key role in human breast cancer etiology and progression (Henderson *et al*., 1988). In breast cancer, endocrine therapies based on antiestrogens and aromatase inhibitors are commonly used to counteract estrogen action (O'Regan and Jordan, 2002). However, the initial response often does not last because tumors frequently develop resistance to hormonal manipulation. Therefore, one of the main clinical challenges is to develop new combined targeted therapies for cancers that are insensitive or resistant to endocrine manipulation.

Estrogen activities are mediated via two nuclear estrogen receptors (ERα and ERβ). They belong to a large conserved superfamily of nuclear receptors that function as ligand‐dependent transcription factors (Heldring *et al*., 2007). Their activities rely on interactions with transcription coregulators that are broadly defined as coactivators or corepressors. Several of these coregulators are involved in chromatin remodeling through multiple enzymatic activities including histone deacetylases (HDACs) (McKenna and O'Malley, 2002).

Human HDACs form a large family of 18 members classified in four groups (I–IV) based on sequence homologies (Haberland *et al*., 2009). Class I enzymes (HDAC1, 2, 3 and 8) are nuclear proteins with ubiquitous expression. Class II HDACs are divided in two subgroups. Class IIa HDACs (HDAC4, 5, 7 and 9) have a tissue‐specific pattern of expression and can shuttle between nucleus and cytoplasm, depending on their phosphorylation status. These HDACs are mainly involved in tissue differentiation (Parra, 2015). Class IIb includes HDAC10 and HDAC6, which is described as a major cytoplasmic regulator. Class III comprises seven sirtuins with a catalytic site that is structurally different from that of the other three classes. Finally, class IV includes only HDAC11.

It has been shown that HDACs affect estrogen signaling at various levels (for review, see (Linares *et al*., 2011)). For instance, HDAC1 interacts with ERα *in vitro* and *in vivo* and suppresses ERα transactivation (Kawai *et al*., 2003). Other studies revealed that *ERS1* gene transcription is regulated by epigenetic modifications and that HDAC inhibitors (HDI) treatment can induce ER expression in ER‐negative breast cancer cell lines (Thomas and Munster, 2009), although this has been questioned more recently (de Cremoux *et al*., 2015). The role of epigenetic modifications (including histone acetylation and DNA methylation) in acquired tamoxifen resistance has also been reported *in vivo* using a preclinical rat model (Hilakivi‐Clarke *et al*., 2017). Interestingly, combinations of HDIs (vorinostat, panobinostat or entinostat) and hormone therapy, such as tamoxifen, letrozole or exemestane, have been used in metastatic breast cancers leading to an improved response (Munster *et al*., 2011; Tan *et al*., 2016; Yeruva *et al*., 2018).

We recently reported that *HDAC9* mRNA expression is markedly increased in the most aggressive breast cancer cell lines, and that HDAC9 expression deregulation (ectopic expression and knockdown) in breast cancer cells significantly alters gene expression, cell proliferation and response to HDIs (Lapierre *et al*., 2016). Here, we investigated HDAC9 role in ERα signaling in breast cancer cells and its expression in antiestrogen‐resistant breast cancer cell lines. Our results demonstrate that HDAC9 overexpression in Michigan Cancer Foundation‐7 (MCF7) breast cancer cells modulates ERα expression and activity and alters ERα ligand effect on cell proliferation. Moreover, HDAC9 expression was markedly deregulated in antiestrogen‐resistant mammary tumor cell lines. Similarly, in human breast tumor samples, a meta‐analysis revealed that high HDAC9 mRNA levels were associated with decreased overall survival in tamoxifen‐treated patients. Altogether, this work highlights strong interactions between class IIa HDAC9 and ERα signaling, suggesting a key role of HDAC9 in antiestrogen resistance of breast cancers.

## 2 Materials and methods

### 2.1 Plasmids and reagents

Estradiol‐17β (E2) was purchased from Sigma‐Aldrich (St Quentin, France). 4‐hydroxy‐tamoxifen (OHTam) and ICI 182 780 (ICI) were kindly donated by Sanofi‐Aventis and AstraZeneca, respectively. Zeocin was from InvivoGen (Toulouse, France). The FLAG‐tagged full length HDAC9 (HDAC9FL) plasmid was a kind gift from A. Zelent (Petrie *et al*., 2003). For stable transfections, the pcDNA3.1‐HDAC9‐zeo plasmid was obtained by subcloning HDAC9FL in the pcDNA3.1zeo vector (Invitrogen, Cergy Pontoise, France) that confers zeocin resistance. The ERα promoter luciferase reporter plasmid was from B. Westley (Donaghue *et al*., 1999). The 17EBLuc+ luciferase reporter plasmid contains one copy of the consensus estrogen‐responsive element (ERE). The pRL‐CMV vector (Promega, Charbonnières, France) was used for normalization.

### 2.2 Cell lines and culture

The MCF7 breast cancer cell line was obtained from the American Type Culture Collection (LGC Standards, Molsheim, France) and was grown using the recommended culture conditions. The OHTam‐resistant MCF7 (MCF7‐OHTR) cell lines have been described previously (Brünner *et al*., 1993; Badia *et al*., 2000; Eric Badia *et al*., 2015) and were authenticated by PCR‐single‐locus technology (Eurofins, Ebersberg, Germany). T47D TamR cells were recently described elsewhere (Mishra *et al*., 2018). MCF7‐OHTR cells were transitory transfected with small interference RNA directed to HDAC9 (Dharmacon, Lafayette, CO, USA). Control and MCF7 cells that stably overexpress HDAC9 were obtained by transfecting MCF7 cells with pcDNA3.1‐zeo and pcDNA3.1‐HDAC9‐zeo respectively, and were isolated on the basis of their zeocin resistance (0.5% zeocin in Dulbecco's modified Eagle's medium supplemented with 10% FBS). All transfections were carried out using Lipofectamine 2000 (Invitrogen) as recommended by the manufacturer. Cells classified as ERα‐positive (MCF7, T47D) or ERα‐negative (MDA‐MB231, MDA‐MB436) were grown using the recommended culture conditions (Lapierre *et al*., 2016).

### 2.3 Cell proliferation

Cells were seeded in triplicate at a density of 2 × 10^3^ cells per well. At the indicated time, 3‐(4,5‐dimethylthiazol‐2‐yl)‐2,5‐diphenyltetrazolium bromide (MTT; Sigma‐Aldrich®, St Louis, MO, USA) was added at 37 °C for 4 h. Formazan crystals were solubilized in DMSO and absorbance read at 560 nm on a spectrophotometer. The measured absorbance correlates with the number of viable cells. Results were normalized to absorbance at day 0. Stripping of endogenous steroids and incubation with ER ligands were done as previously described (Duong *et al*., 2008). MCF7 and MCF7‐OHTR cells (transfected or not with siHDAC9) were seeded at a density of 2500 cells per well into E‐Plate 16 (ACEA Biosciences, Inc., San Diego, CA, USA) containing 150 μL of medium supplemented with 10% FBS per well. Dynamic monitoring of cell growth was performed every 24 h during 10 days using the impedance‐based xCELLigence system (ACEA Biosciences, Inc.). The cell index was derived from measurement of cell‐electrode impedance which correlates with the number of viable cells.

### 2.4 Luciferase reporter assays

Cells were plated in 96‐well plates (10^5^ cells per well) in triplicate 24 h before DNA transfection (250 ng of total DNA) using the Jet PEI (Polyplus, Illkirch, France) method. Firefly luciferase values were measured and normalized to the Renilla luciferase activity [pRL‐CMV‐Renilla plasmid (Ozyme®, Montigny‐le‐Bretonneux, France)]. Values were expressed as the mean ratio of luciferase activities.

### 2.5 Western blot analysis

Whole cell proteins were extracted as previously described (Duong *et al*., 2008); 30 μg protein extracts were separated by SDS/PAGE and analyzed by western blotting using primary polyclonal antibodies against ERα (clone sc‐543; Tebu‐bio, Le Perray‐en‐Yvelines, France) or actin (Sigma) and the Chemiluminescence Reagent Plus kit (Perkin‐Elmer Life Science, Villebon S/Yvette, France).

### 2.6 Immunofluorescence staining

Breast cancer cells were fixed in 4% formaldehyde, permeabilized with 1% Triton X‐100 and saturated with 1% BSA at room temperature for 3 h. Cells were incubated first with an anti‐HDAC9 antibody (Abcam, Cambridge, UK, 18970; 1 : 100 in PBS with 1% BSA) and then with an Alexa‐conjugated secondary antibody (Invitrogen). After washing, cells were counterstained with Hoechst (Sigma‐Aldrich, St Quentin, France) and mounted for fluorescence analysis.

### 2.7 RNA extraction and real‐time quantitative PCR

Total RNA extraction and reverse transcription were performed as previously described (Duong *et al*., 2008). Real‐time PCR quantification was done on a LC480 instrument using LightCycler 480 SYBR Green PCR Master (Roche Diagnostics, Meylan, France). For each sample, the expression levels of the target genes were normalized to the mean levels of the housekeeping genes TATA‐binding protein (*TBP*) and ribosomal protein S9, and expressed relative to a calibrator sample. The primers for the *HDAC1* to *HDAC11*, estrogen receptor 1 (*ESR1*) and *TBP* genes have been described elsewhere (Annicotte *et al*., 2006; Chen *et al*., 2004; Delfour *et al*., 2006; Duong *et al*., 2008). Other primers used in this study are available upon request.

### 2.8 Breast tumor samples analysis

The datasets used to analyze HDAC9 expression in breast cancers [GSE26459 (Gonzalez‐Malerva *et al*., 2011), E‐TABM‐158 (Chin *et al*., 2006), GSE27473 (Al Saleh *et al*., 2011), GSE47561 (Ur‐Rehman *et al*., 2013) and the Fan dataset (Fan *et al*., 2006)] are listed in Table S1. The Oncomine Platform (https://www.oncomine.org) was used to investigate HDAC9 expression in the different breast cancer datasets grouped by ERα status. The BreastMark algorithm (http://glados.ucd.i.e/BreastMark), developed by Molecular Therapeutics for Cancer, Ireland, was used for Kaplan–Meier analysis of several datasets (Madden *et al*., 2013). The gene set enrichment analysis (GSEA) was implemented with the GSEA Java application (http://software.broadinstitute.org/gsea/index.jsp) (Subramanian *et al*., 2005). GSEA of the ISDB3008 dataset was performed as previously described (Gao *et al*., 2014).

### 2.9 Global gene expression analysis

Global transcriptome analysis of OHTam‐sensitive and OHTR and HDAC9‐overexpressing and control MCF7 cells was performed using human oligonucleotide HG‐U219 microarrays (ThemoFisher Scientific, Waltham, MA, USA) processed at the Microarray Core Facility of the Institute for Regenerative Medicine and Biotherapy (IRMB), CHRU‐INSERM‐UM Montpellier (http://www.chu-montpellier.fr/fr/irmb/). After image processing with the Affymetrix GeneChip command console, the CEL files were analyzed using the affymetrix expression console™ (ThemoFisher Scientific) Software v1.3.1 and normalized with the robust multi‐array average algorithm. Gene annotation was performed using NetAffx (http://www.affymetrix.com; October 2014). The significant analysis of microarrays (Stanford, CA, USA) software with the Wilcoxon test and 300 sample label permutations was used to identify differentially expressed genes relative to control. Hierarchical clustering analyses based on the expression levels of the differentially expressed genes were performed by using the cluster and treeview packages (Eisen *et al*., 1998). Gene Ontology (GO) biological process enrichment analyses of the differentially expressed genes were generated with the ingenuity pathway analysis (IPA) (Germantown, MD, USA) software. Selected genes were imported in the IPA database and were categorized on the basis of their biological process and molecular functions (www.ingenuity.com).

### 2.10 Statistical analysis

Results are expressed as the mean ± standard deviation (SD). Data comparison between groups was performed using the Student *t*‐test or Mann–Whitney *U*‐test. A probability level of 0.05 was chosen for statistical significance. Statistical analyses were performed using graphpad prism 6, version 6.01 (GraphPad Software, San Diego, CA, USA).

## 3 Results

### 3.1 HDAC9 inhibits ERα expression and activity in MCF7 breast cancer cells

**Figure 1:**
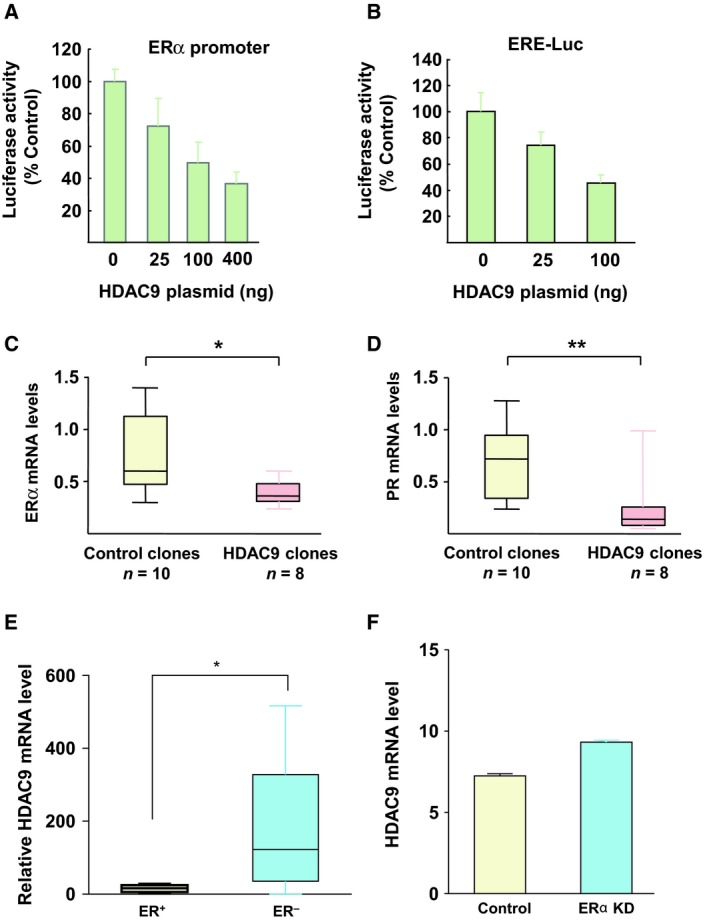
Cross talk between HDAC9 and ERα signaling in breast cancer cells. (A) MCF7 cells were cotransfected with a 4‐kb fragment of the ERα promoter and increasing concentrations of full length HDAC9. Results represent the luciferase activity measured after normalization to Renilla luciferase activity and relative to the values obtained in cells not transfected with the HDAC9 plasmid (control). Data are the mean ± SD of triplicate wells and are representative of two independent experiments. (B) The same as in panel A, but with cells cotransfected with an ERE‐luciferase reporter plasmid and increasing concentrations of full length HDAC9. (C) Total RNA was extracted from control (empty vector; *n* = 10) and HDAC9‐overexpressing cell clones (*n* = 8). ERα mRNA levels were quantified using RT‐qPCR. Results represent the mean FC ± SD vs control MCF7 cells; *P < 0.05, **P < 0.01 (Mann–Whitney test). (D) The same as in panel C, but for *PGR* mRNA. (E) Total RNA was extracted from ERα‐positive or ERα‐negative breast cancer cell lines and ERα mRNA levels were quantified using RT‐qPCR; *P < 0.05. (F) HDAC9 expression levels in parental and MCF7 cells with silenced ERα expression (205659_at probe set) were extracted from the GEO profile GSE27473 and compared (Al Saleh *et al*., 2011).

A previous work showed that class IIa HDAC9 negatively regulates ERα expression *in vivo* in mouse heart and in neonatal rat cardiomyocytes (van Rooij *et al*., 2010). Here, to determine whether HDAC9 also modulates ERα signaling in breast cancer cells, a luciferase reporter assays was used to test the activity of a 4‐kb fragment of the ERα promoter in MCF7 breast cancer cells transfected or not with a plasmid that expresses HDAC9FL. HDAC9 significantly decreased ERα promoter activity in a dose‐dependent manner (Fig. 1A). Similarly, HDAC9 overexpression inhibited ERE‐mediated transcriptional activation in MCF7 cells (Fig. 1B). Altogether, these data suggested that HDAC9 represses estrogen signaling in breast cancer cells.

### 3.2 Inhibition of ERα signaling in MCF7 breast cancer cells that stably overexpress HDAC9

To better characterize HDAC9 effect on estrogen signaling in breast cancer cells, two ERα‐positive MCF7 cell clones that stably express HDAC9FL (MCF7‐HDAC9FL, clones 4‐1 and 1‐1) were selected and characterized relative to control clones (P1 and P2; empty vector). HDAC9 mRNA and protein levels were higher in MCF7‐HDAC9FL than in control clones (~ 4.8 and ~ 8.5 times, respectively, for mRNA levels; Fig. S1A,B). Moreover, in agreement with the luciferase reporter assay results, the mRNA levels of endogenous ERα (Fig. 1C) and its target progesterone receptor (PR; Fig. 1D) were significantly decreased in MCF7‐HDAC9FL cells compared with controls (mean ± SD for ERα: 0.38 ± 0.12 and 0.74 ± 0.37 respectively, *P* = 0.02; and for PR: 0.24 ± 0.32 and 0.71 ± 0.36 respectively, *P* = 0.007). Western blot analysis confirmed the marked decrease of ERα expression in MCF7‐HDAC9FL cells (Fig. S2A). Finally, GSEA of microarray data obtained from an independent pool of MCF7 cell clones that overexpress or not HDAC9 (Lapierre *et al*., 2016) confirmed that HDAC9 overexpression led to a significant decrease of genes belonging to the ERα signaling pathway (Fig. S2B). Altogether, these results demonstrated that HDAC9 inhibits ERα expression and activity in human breast cancer cells.

### 3.3 ERα inhibits HDAC9 expression in human breast cells and tumors

We previously reported that HDAC9 expression was significantly lower in ERα‐positive breast cancer cell lines, such as MCF7 and T47D, compared with ERα‐negative breast cancer cell lines, such as MDA‐MB231 and MDA‐MB436 (Fig. 1E) (Lapierre *et al*., 2016). Interestingly, the same inverse correlation was found by analyzing several human breast tumor datasets, including the E‐TABM‐158 breast cancer dataset (Chin *et al*., 2006). In this cohort, HDAC9 mRNA levels were significantly higher in ERα‐negative than in ERα‐positive breast tumors (*P* < 0.0001; Fig. S2C). Similar findings were obtained by analysis of the NKI (Fan *et al*., 2006) and the GSE47561 datasets (Ur‐Rehman *et al*., 2013) (data not shown). Moreover, meta‐analyses performed using the breast cancer gene‐expression miner v3 tool (expression and correlation analysis modules) (Jézéquel *et al*., 2013) also showed an inverse correlation between ERα and HDAC9 expression (Fig. S2D) as well as between HDAC9 and PR expression in breast tumors (Fig. S2E).

Finally, analysis of the GSE27473 dataset (Al Saleh *et al*., 2011) indicated that ERα silencing in MCF7 cells resulted in a significant increase of HDAC9 mRNA levels (Fig. 1F). Conversely, ectopic expression of ERα in ER‐negative cells reduced HDAC9 mRNA levels (Fig. S2F). Altogether, these data indicated that ERα loss is involved in the increased *HDAC9* gene expression detected in ER‐negative breast cancer cells.

### 3.4 HDAC9 expression in antiestrogen‐sensitive and antiestrogen‐resistant breast cancer cells

**Figure 2:**
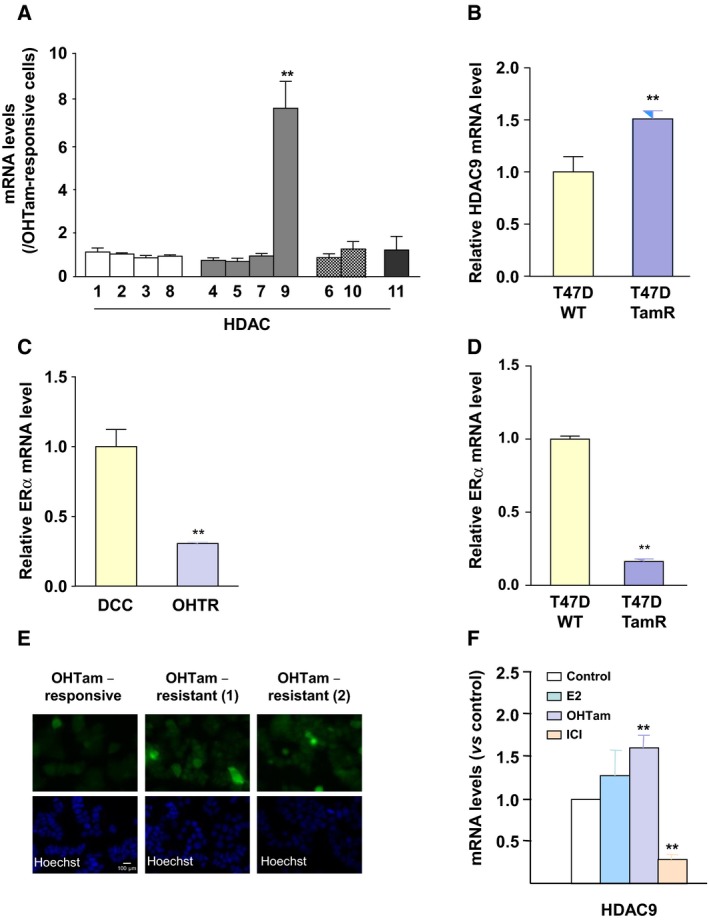
HDAC9 expression in antiestrogen‐resistant breast cancer cells. (A) Total RNA was extracted from OHTam‐sensitive and OHTam‐resistant MCF7 cell lines and *HDAC* mRNA levels were quantified by RT‐qPCR. Results represent the mean ± SD of three independent cell cultures and are expressed relative to the *HDAC* mRNA levels of OHTam‐responsive cells, used as reference; **P < 0.01 (Mann–Whitney test compared with OHTam‐responsive cells). (B) HDAC9 mRNA levels were quantified by RT‐qPCR in T47D TamR cells (Mishra *et al*., 2018). Results are expressed relative to the HDAC mRNA levels in parental cells and represent the mean ± SD of five independent cell cultures; **P < 0.001 (*t*‐test compared with parental T47D cells). (C) Total RNA was extracted from OHTam‐responsive (DCC) and resistant (OHTR) MCF7 cells and ERα mRNA levels were quantified by RT‐qPCR. Results represent the mean ± SD of three independent cell cultures and are expressed relative to the ERα mRNA levels in DCC cells, used as reference; **P < 0.01 (Mann–Whitney test compared with DCC cells). (D) The same as in panel B for the quantification of ERα mRNA levels by RT‐qPCR in T47D TamR cells. (E) HDAC9 expression was analyzed by immunofluorescence in OHTam‐responsive MCF7 cells and in two sets of OHTam‐resistant MCF7 cells (1 and 2) using an anti‐HDAC9 antibody (top panel); nuclei were stained with Hoechst (bottom panel). Scale bar corresponds to 100 μm. (F) MCF7 cells were incubated with 10^−8^ M E2, OHTam, ICI or solvent alone (EtOH; Control) for 20 h and then HDAC9 mRNA levels were quantified by RT‐qPCR. Results are expressed relative to the HDAC mRNA levels in control cells and represent the mean ± SD of three independent cell cultures; **P < 0.01 (Mann‐Whitney test compared with control cells).

As ERα expression is lost or decreased in OHTR cells (Al Saleh *et al*., 2011), we next asked whether breast cancer cell resistance to OHTam could be associated with changes in *HDAC9* gene expression. Comparison of the HDAC mRNA profile in previously described MCF7 cell lines that are responsive or resistant to OHTam (Badia *et al*., 2000, 2015) showed that among the 11 HDACs analyzed, only HDAC9 expression was significantly higher (7.6 times) in OHTR than in parental OHTam‐responsive cells (Fig. 2A). Similar results were obtained with OHTR T47D (Mishra *et al*., 2018) (Fig. 2B) as well as in other MCF7 cell lines (Brünner *et al*., 1993; Eric Badia *et al*., 2015; Gonzalez‐Malerva *et al*., 2011) (Fig. S3A,B). As expected, HDAC9 mRNA overexpression in OHTR cells was associated with decreased ERα levels (Figs 2C,D and S3C). HDAC9 overexpression in OHTR MCF7 cells was confirmed also by immunofluorescence analysis (Fig. 2E).

Then, to determine whether HDAC9 expression could be regulated by short‐term treatments with estrogens and antiestrogens, class I, II and IV HDAC mRNA levels were quantified in ERα‐positive MCF7 cells incubated with E2 or with the antiestrogens OHTam and ICI for 24 h. These ER ligands affected significantly only the expression of several class IIa HDACs, including HDAC9 (Figs 2F and S3D–G). Specifically, the pure antagonist ICI had a strong inhibitory effect on HDAC9 gene expression, while OHTam, and to a lesser extent, E2 had a significant positive effect (Fig. 2F).

### 3.5 Effect of HDAC9 on ER‐dependent cell proliferation

**Figure 3:**
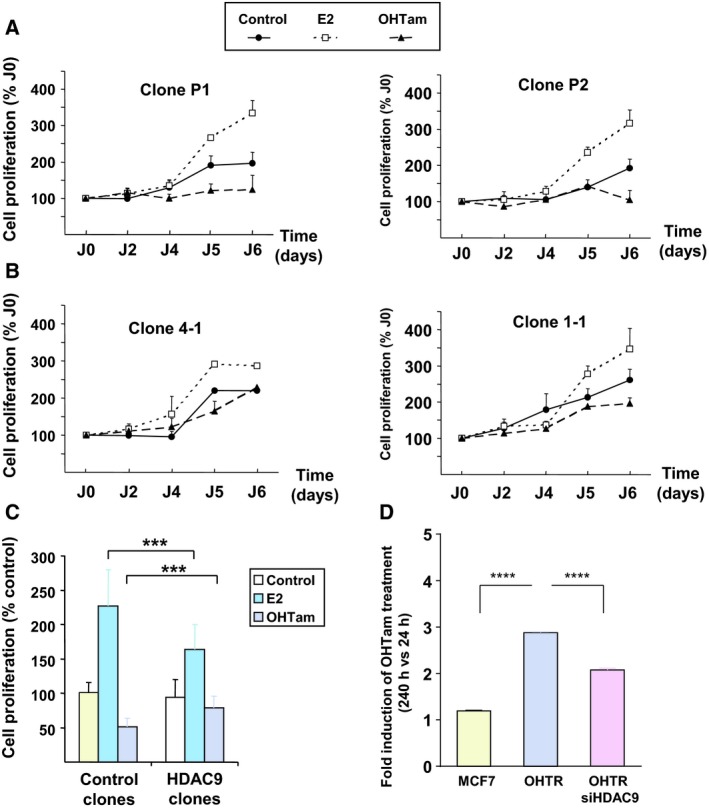
HDAC9 and the regulation of breast cancer cell proliferation by ER ligands. (A) Control MCF7 cell clones (empty vector; P1 and P2) were cultured in DCC medium supplemented with E2, OHTam or solvent alone (Control) for 5 days. Cell proliferation was measured with the MTT assay at days 2, 4, 5 and 6 and expressed relative to the absorbance at day 0. Results are the mean ± SD of three independent experiments, performed in triplicates. (B) The same as in A, but with MCF7‐HDAC9 cell clones (4‐1 and 1‐1). (C) Data obtained for each condition (control and MCFT‐HDAC9 cell clones) were pooled and compared. Data represent the mean ± SD of three independent experiments for each clone; ***P < 0.001 (Mann–Whitney test). (D) The growth of MCF7 and MCF7‐OHTR cells (transfected or not with a siRNA directed against HDAC9) was monitored using the XCELLigence system for a total duration of 10 days. The cell index corresponding to the number of viable cells was determined. The results represent, for each condition, cell proliferation measured after 240 h of culture in the presence of OHTam normalized by the values measured at 24 h (means ± SD; two independent experiments). ****P < 0.0001 (Mann‐Whitney test).

To investigate whether HDAC9 overexpression modulated ER‐dependent cell proliferation, MCF7‐HDAC9FL (4‐1 and 1‐1) and control clones (P1 and P2) were cultured in the presence of E2 or OHTam. MCF7‐HDAC9FL cells still responded to estrogen stimulation, although to a lesser extent than controls, but remarkably could proliferate also in the presence of the antiestrogen OHTam (Fig. 3A,B). Quantification of the results from independent cell cultures confirmed that MCF7‐HDAC9FL cells were significantly less sensitive to estrogen stimulation and to OHTam inhibition than controls (*P* < 0.001; Fig. 3C).

To emphasize these results, we then knocked down HDAC9 expression in MCF7‐OHTR cells and monitored the effect on OHTam resistance. Using a siRNA approach, HDAC9 expression was efficiently silenced in MCF7‐OHTR cells leading to HDAC9 mRNA levels comparable to that of MCF7 cells (Fig. S4). In the presence of OHTam, parental MCF7 cells almost did not grow reflecting their sensitivity to the inhibition of E2 signaling. By contrast, MCF7‐OHTR cell number significantly increased almost by threefold during the same period of time confirming their resistance to OHTam. Interestingly, when treated with the HDAC9 siRNA, MCF7‐OHTR cells grew significantly less in the presence of OHTam (Fig. 3D), strongly suggesting that silencing HDAC9 expression decrease their resistance to OHTam treatment. Altogether, these data clearly supported a link between HDAC9 overexpression and antiestrogen resistance in breast cancer cells.

### 3.6 Identification of genes coregulated by HDAC9 and resistance to antiestrogens

In order to determine whether HDAC9 overexpression and resistance to OHTam led to the deregulation of common genes, a global transcriptome analysis of MCF7 cells sensitive and resistant to OHTam was performed and compared to that performed in MCF7‐Control and MCF7‐HDAC9FL cells (Lapierre *et al*., 2016).

**Figure 4:**
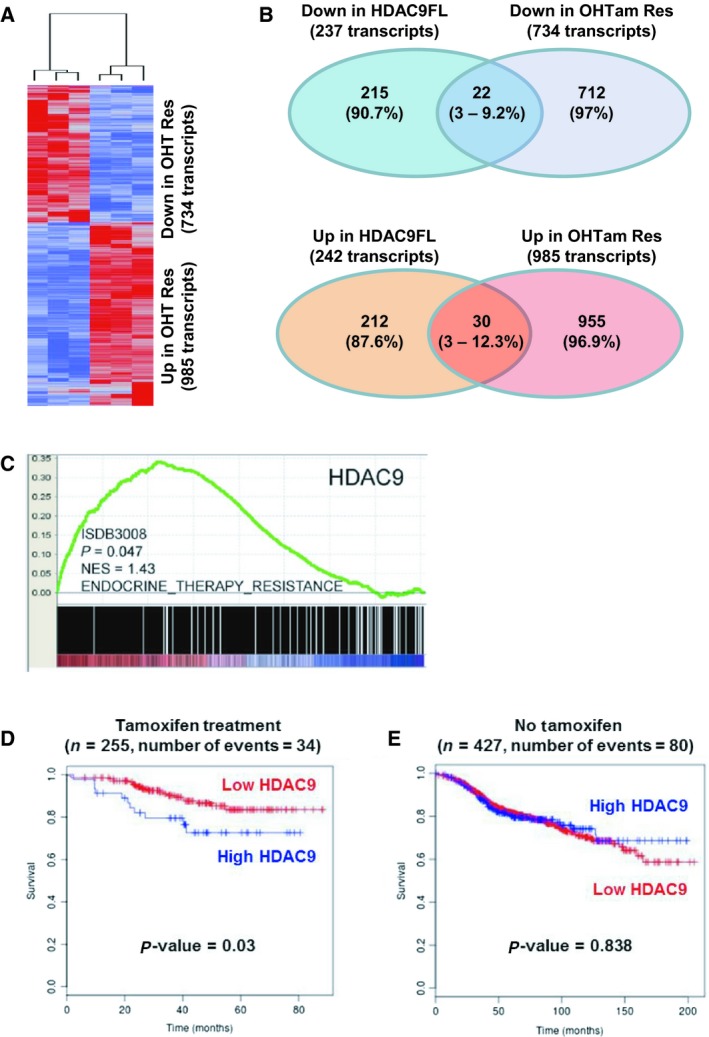
Transcriptomic analyses in MCF7 cells and expression in human breast cancer tumors. (A) The molecular signatures of OHTam‐sensitive and OHTam‐resistant MCF7 cells were visualized by hierarchical clustering of the 1719 genes that are differentially expressed in the two conditions (genes are arranged in rows and samples in columns). For each condition, a tree represents the relationship among samples and the branch lengths reflect the degree of similarity between samples according to the gene expression profile. In red, upregulated genes; in blue, downregulated genes. (B) Schematic representation showing the overlap between downregulated (top drawing) and upregulated (bottom drawing) transcripts in MCF7‐HDAC9FL and OHTam‐resistant MCF7 cells. The number (percentage) of transcripts deregulated in each condition is indicated. (C) GSEA of *HDAC9* mRNA expression relative to the endocrine therapy resistance gene set in breast cancer (*n* = 2795 samples). (D) Kaplan–Meier analysis with the BreastMark algorithm of survival in patients with breast cancer treated with tamoxifen (*n* = 255 tumor samples) relative to HDAC9 expression using a low cutoff analysis (lower quartile). Disease‐free survival was considered as the survival end point and if not available, distant disease‐free survival or overall survival was used. Significance was calculated using the log rank test. (E) The same as in panel D, but for untreated patients (*n* = 427 tumor samples).

Comparison of the expression profiles of OHTam‐sensitive and OHTR MCF7 cells identified 1719 transcripts that were differentially expressed with a fold change (FC) ≥ 2 and a false discovery rate ≤ 0.05 (734 transcripts were downregulated and 985 transcripts upregulated in OHTR cells; see Table S2 for the list of the top up‐ and downregulated genes). Hierarchical clustering of OHTam‐sensitive and OHTR MCF7 cells based on the expression profile of the 1719 transcripts (Fig. 4A) showed that all OHTam‐sensitive MCF7 cell samples clustered in one branch and all OHTR samples in another branch. The GO biological process enrichment analysis of these transcriptomic data with IPA revealed that in resistant cells, the most significantly upregulated biological process was ‘Cell death and survival’, whereas ‘Cellular growth and proliferation’ was the most significantly downregulated process (Table S3).

The transcriptomic analysis of MCF7‐Control and MCF7‐HDAC9FL cells (Lapierre *et al*., 2016) identified 237 probe sets that corresponded to genes downregulated in MCF7‐HDAC9FL cells (22 also downregulated in OHTR‐MCF7 cells) and 242 probe sets corresponding to genes upregulated in MCF7‐HDAC9FL cells (30 also upregulated in OHTR cells; Fig. 4B). The list of genes similarly regulated by HDAC9 overexpression and OHTam resistance is in Table S4. *GFRA1*,* SGK3*, nuclear receptor interacting protein 1 (*NRIP1*), *RAB31* and *SLC39A6* were among the most represented downregulated genes, whereas *MUC1* and, to a lesser extent, *MAGED2* were represented more than once in the list of common upregulated genes. Particularly, *ESR1* and some of its target genes, such as *NRIP1* (Augereau *et al*., 2006) and *SGK3* (Wang *et al*., 2011), were downregulated in both conditions, in agreement with the data in Figs 1, 2, 3.

Moreover, GSEA (Subramanian *et al*., 2005) performed using the transcriptomic data of MCF7‐Control and MCF7‐HDAC9FL cells (Lapierre *et al*., 2016) showed that gene sets related to tamoxifen resistance, including ‘Endocrine Therapy Resistance’ and ‘Tamoxifen Resistance DN’ were differentially represented (*P* < 0.05) in MCF7‐HDAC9FL samples compared with MCF7‐Control (Fig. S5). This supports the hypothesis that HDAC9‐regulated genes are involved in antiestrogen resistance.

### 3.7 Prognostic value of HDAC9 expression in human breast tumors

To investigate the putative role of HDAC9 expression in the patient response to endocrine therapies, GSEA was performed using expression data from the ISDB3008 dataset (2795 breast cancer samples), as already described (Gao *et al*., 2014). This analysis indicated that HDAC9 expression was positively correlated with genes overexpressed in tumor samples from patients resistant to endocrine therapy (Fig. 4C, *P* = 0.047), confirming the data shown in Fig. S4.

Finally, HDAC9 expression prognostic value was determined using the BreastMark database (Madden *et al*., 2013) that allows the reanalysis of 26 different transcriptomic datasets to correlate the clinical outcome with differential gene expression levels and to define the prognostic potential of a given gene. The analysis was restricted to patients with tumors being estrogen and PR‐positive and HER2‐negative. HDAC9 levels were of significant prognostic value, and high levels were correlated with shorter overall survival in tamoxifen‐treated (*n* = 255; *P* = 0.03; Fig. 4D), but not in untreated patients (*n* = 427; *P* = 0.838; Fig. 4E). Altogether, these results supported our *in vitro* data and demonstrated the prognostic value of HDAC9 levels in patients with breast cancer who received antiestrogen therapy.

## 4 Discussion

**Figure 5:**
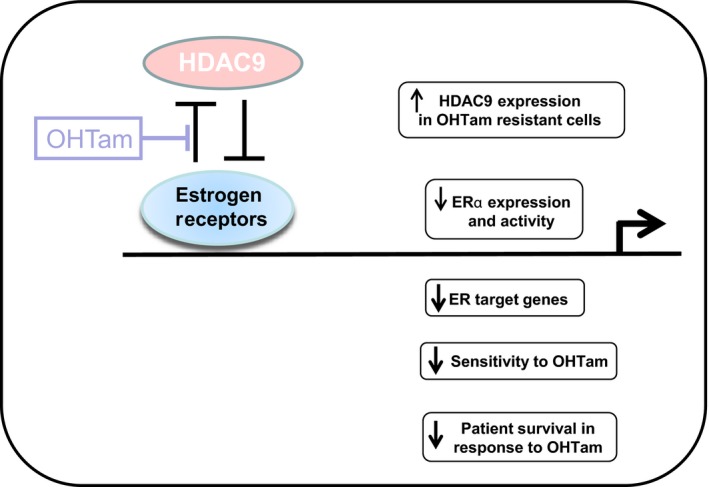
Effect of HDAC9 on estrogen signaling and tamoxifen response in breast cancer cells. Schematic model of the putative role of HDAC9 in estrogen signaling in human breast cancer cells. HDAC9 expression is inversely correlated with ERα expression in breast cancer and is upregulated in OHTam‐resistant breast cancer cells. HDAC9 decreases ERα expression and activity, ER target gene expression, OHTam cytotoxicity and survival of patients treated with tamoxifen.

Previous studies have underlined the potential role of HDACs in breast tumor progression and their cross talk with ER signaling (Linares *et al*., 2011; Thomas and Munster, 2009). Here, we demonstrated the strong interaction of class IIa HDAC9 with ERα signaling and its implication in OHTam resistance in breast cancer cell lines and tumor samples. These results are schematically summarized in Fig. 5.

Since its cloning (Zhou *et al*., 2001) and thanks to the analysis of knockout (KO) mice, HDAC9 has been involved in various pathophysiological processes including muscle differentiation (Zhang *et al*., 2001), heart response to stress (Zhang *et al*., 2002) or immune regulatory T cell function (de Zoeten *et al*., 2010). HDAC9 plays also an important role in various type of cancers including, among others, oral squamous cell carcinoma (Rastogi *et al*., 2016), retinoblastoma (Zhang *et al*., 2016) and breast cancer (Lapierre *et al*., 2016).

Few years ago, van Rooij *et al*. (2010) reported that class IIa HDAC9 and HDAC5 reduce ERα expression in rat cardiomyocytes via the control of MEF2 transcription factor activity, which could explain ERα cardioprotective effect in HDAC9 KO female mice. Similarly, our study showed that HDAC9 decreases ERα expression (ERα mRNA and protein levels, *ESR1* gene promoter transcription) and transcriptional activity (ERE‐mediated response in luciferase reporter assays and expression of the E2‐regulated *PGR* gene).

Our work also demonstrated that *HDAC9* is overexpressed in different OHTR MCF7 cell lines. The precise mechanisms involved remain to be determined, although loss of ERα expression could contribute to HDAC9 upregulation in antiestrogen‐resistant breast cancer cell lines. Indeed, HDAC9 expression is increased upon ERα silencing in breast cancer cell lines (Al Saleh *et al*., 2011). Moreover, our analysis of publicly available cDNA array data showed a significant increase in *HDAC9* mRNA levels in ERα‐negative tumors compared with ERα‐positive samples, validating the biological relevance of the results obtained in breast cancer cell lines. Other mechanisms could explain *HDAC9* gene upregulation in ER‐negative and in antiestrogen‐resistant breast cancer cells. For instance, comparative genomic hybridization (CGH) array analysis from 532 breast tumors indicated a significant copy number gain (23.7% of the cases) for the 7p21‐p15.3 region that encompasses the *HDAC9* gene (Mahlknecht *et al*., 2002; B. Orsetti, data not shown). Similarly, in cervical carcinoma, the *HDAC9* gene was identified by CGH array in a region of high copy number gain (Choi *et al*., 2007) thus suggesting that an increase in gene copy number could also be involved in HDAC9 gene overexpression. More recently, it was reported that *HDAC9* is hypomethylated in hepatocellular carcinoma (Archer *et al*., 2010) suggesting that epigenetic modifications could represent another way to overexpress HDAC9.

We then showed that HDAC9 overexpression decreases the sensitivity of MCF7 breast cancer cells to ER ligands in proliferation assays. Most importantly, we found that silencing HDAC9 expression in MCF7‐OHTR cells reduced their OHTam resistance. The decreased ERα expression in MCF7 cells upon HDAC9 overexpression, even at moderate levels, may be one of the mechanisms by which deregulated HDAC9 expression is linked to OHTam resistance in ERα‐positive breast tumor cells. In line with this hypothesis, we found that ERα expression was decreased in the various OHTR breast tumor cell lines developed by our group. However, other mechanisms have been involved in breast tumor resistance to OHTam, including activation of growth factor pathways (Riggins *et al*., 2007). The impact of HDAC9 expression on such mechanisms should be further studied to better understand the development of OHTam resistance.

We also observed the association of HDAC9 with OHTam resistance in breast cancer samples. Indeed, HDAC9 expression was positively correlated with genes overexpressed in endocrine therapy‐resistant tumor samples, and had a prognostic significance (shorter overall survival with high HDAC9 expression) only in patients who received OHTam. These data are in accordance with our *in vitro* observations showing that HDAC9 overexpression reduces OHTam antiproliferative effect in breast cancer cell lines. Moreover, in our global transcriptome analyses, we identified several genes that are similarly deregulated in OHTR cells and HDAC9‐overexpressing cells. For instance, we confirmed the downregulation of the *SMC3* gene (Mendes‐Pereira *et al*., 2012) and the overexpression of the *MUC1* (Kharbanda *et al*., 2013) and *S100P* (Zhou *et al*., 2012) genes in OHTR cells and demonstrated, for the first time, their regulation also by HDAC9 overexpression. Altogether, these results argue for an important role of HDAC9 overexpression in the acquisition of tamoxifen resistance by breast cancer cells.

## 5 Conclusions

Antiestrogen‐based endocrine therapies are commonly used to treat ER‐positive breast cancers, but tumors usually become resistant to such hormonal manipulation. Our study underlines strong interactions between class IIa HDAC9 and ERα signaling (Fig. 5) and undercovers the key role of HDAC9 in antiestrogen resistance of breast cancer cells and tumors. Our data suggest that HDAC9 might represent a new marker of antiestrogen response. Moreover, based on our results, it appears likely that targeting HDAC9 might represent a novel therapeutic target to treat endocrine therapy‐resistant cancers.

## Conflict of interest

The authors declare no conflict of interest.

## Author contributions

VC and NB designed and supervised the study; AL, ML, ET, CD, CF and AB performed the experiments; SA, GT and JJ performed the bioinformatic analyses; OS and EB provided reagents; AL, ML, NB and VC analyzed the data; ML, NB and VC wrote the manuscript.

## Supporting information


**Fig. S1**. Characterization of HDAC9‐overexpressing MCF7 cell clones.
**Fig. S2**. Expression of HDAC9 mRNA in breast cancer cells and tissue samples.
**Fig. S3**. Expression of HDAC9 mRNA in antiestrogen‐resistant cells.
**Fig. S4**. SiRNA‐mediated knockdown of HDAC9 expression in MCF7‐OHTR cells.
**Fig. S5**. GSEA of genes that are differentially regulated by HDAC9 in breast cancer cells.
**Table S1**. List of the different datasets reanalyzed with the corresponding reference and the main observation obtained from their use.
**Table S2**. List of the most deregulated genes in OHTR vs OHTam‐sensitive MCF7 breast cancer cells.
**Table S3**. List of the most deregulated GO biological profiles in OHTR vs OHTam‐sensitive MCF7 breast cancer cells.
**Table S4**. List of the most common deregulated genes in OHTR and HDAC9‐overexpressing MCF7 cells.

## Data Availability

The datasets used and/or analyzed during the current study are available from the corresponding author.
