# Supplementary material for: Increased expression of the HDAC9 gene is associated with antiestrogen resistance of breast cancers
Source: Mol Oncol. 2019 Jun 12;13(7):1534–47. doi: 10.1002/1878-0261.12505 (PMC6599838; doi:10.1002/1878-0261.12505)
Supplement: Supplementary file 1 — Fig. S1. Characterization of HDAC9‐overexpressing MCF7 cell clones. Fig. S2. Expression of HDAC9 mRNA in breast cancer cells and tissue samples. Fig. S3. Expression of HDAC9 mRNA in antiestrogen‐resistant cells. Fig. S4. SiRNA‐mediated knockdown of HDAC9 expression in MCF7‐OHTR cells. Fig. S5. GSEA of genes that are differentially regulated by HDAC9 in breast cancer cells. Table S1. List of the different datasets reanalyzed with the corresponding reference and the main observation obtained from their use. Table S2. List of the most deregulated genes in OHTR vs OHTam‐sensitive MCF7 breast cancer cells. Table S3. List of the most deregulated GO biological profiles in OHTR vs OHTam‐sensitive MCF7 breast cancer cells. Table S4. List of the most common deregulated genes in OHTR and HDAC9‐overexpressing MCF7 cells. [file MOL2-13-1534-s001.docx]

**SUPPLEMENTARY INFORMATION FILES**
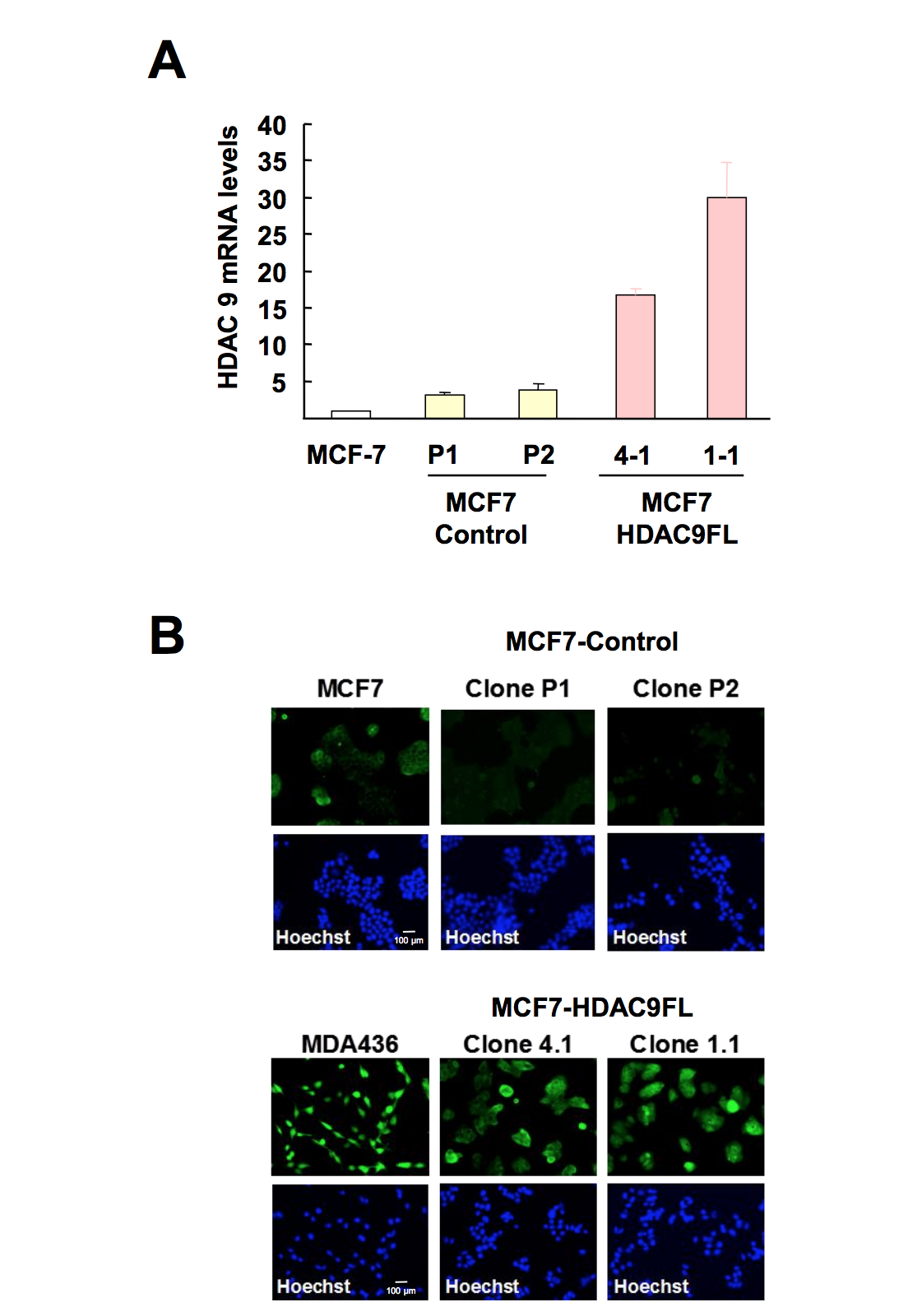


***Supplementary Figure 1: Characterization of HDAC9-overexpressing MCF7 cell clones.***

**(A)** MCF7 cells were stably transfected with vector alone (clones P1 and P2) or full length HDAC9 plasmid (clones 4-1 and 1-1). *HDAC9* mRNA levels were quantified by RT-qPCR. Results are expressed relative to the *HDAC* mRNA levels in non-transfected MCF7 cells. **(B)** MCF7 cells, MDA436 cells, control (P1 and P2) and MCF7-HDAC9FL cell clones (clones 1-1 and 4-1) were analyzed by immunofluorescence using an anti-HDAC9 antibody (top panel) and Hoechst nuclear staining (bottom panel). Scale bars correspond to 100 µm.

***
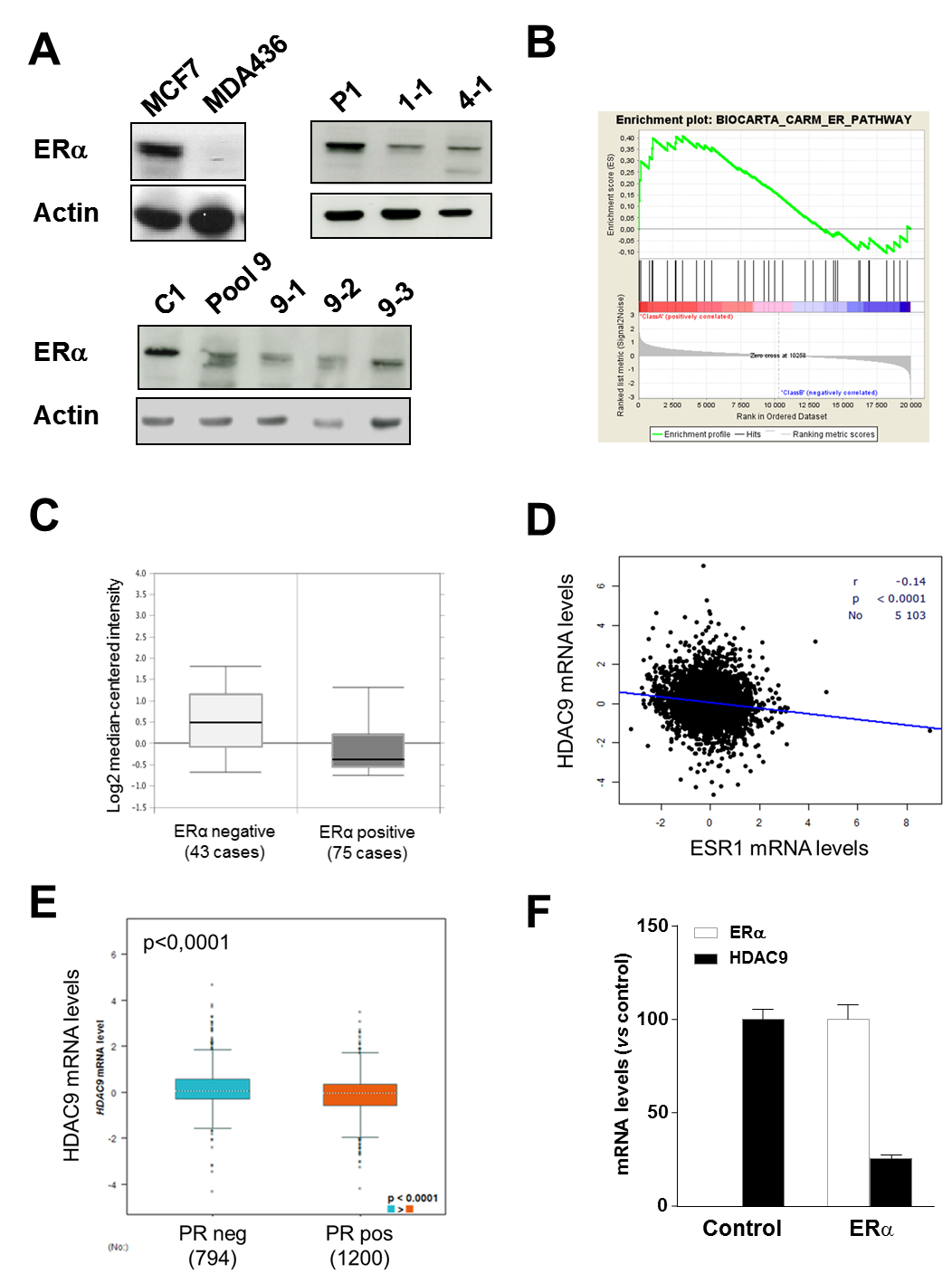
***

***Supplementary Figure 2: Expression of HDAC9 mRNA in breast cancer cells and tissue samples.***

**(A)** ERα expression was analyzed in total protein extracts from MCF7 and MDA436 cells, control clones (P1 and C1) and MCF7-HDAC9FL clones or pool (pool 9, clones 1-1, 4-1, 9-1, 9-2, and 9-3) by western blotting using an anti-ERα antibody. Actin was used as loading control. **(B)** GSEA analysis of the BIOCARTA_CARM_ER_pathway showing a significant enrichment (p<0.05) among the genes downregulated in the previously described MCF7-HDAC9FL vs MCF7-Control cells (Lapierre *et al.*, 2016). **(C)** The Oncomine database was used to investigate the expression of HDAC9 (205659_at probe set) in the E-TABM-158 breast cancer dataset (28) grouped according to their ERα status (43 negative and 75 positive samples) (p<0.0001). **(D)** Correlation between *HDAC9* and *ESR1* mRNA levels in 5103 breast cancer samples using the Breast Cancer Gene-Expression Miner v3 tool. The correlation coefficient and the p value are indicated. **(E)** The Breast Cancer Gene-Expression Miner v3 tool was used to assess HDAC9 expression levels in breast tumors that express (1200 samples) or not (794 samples) the *PGR* gene. The p-value is indicated. **(F)** ER-negative cells were transiently transfected with an ERα expression vector, and the mRNA levels of ERα and HDAC9 were quantified by RT-qPCR.

***
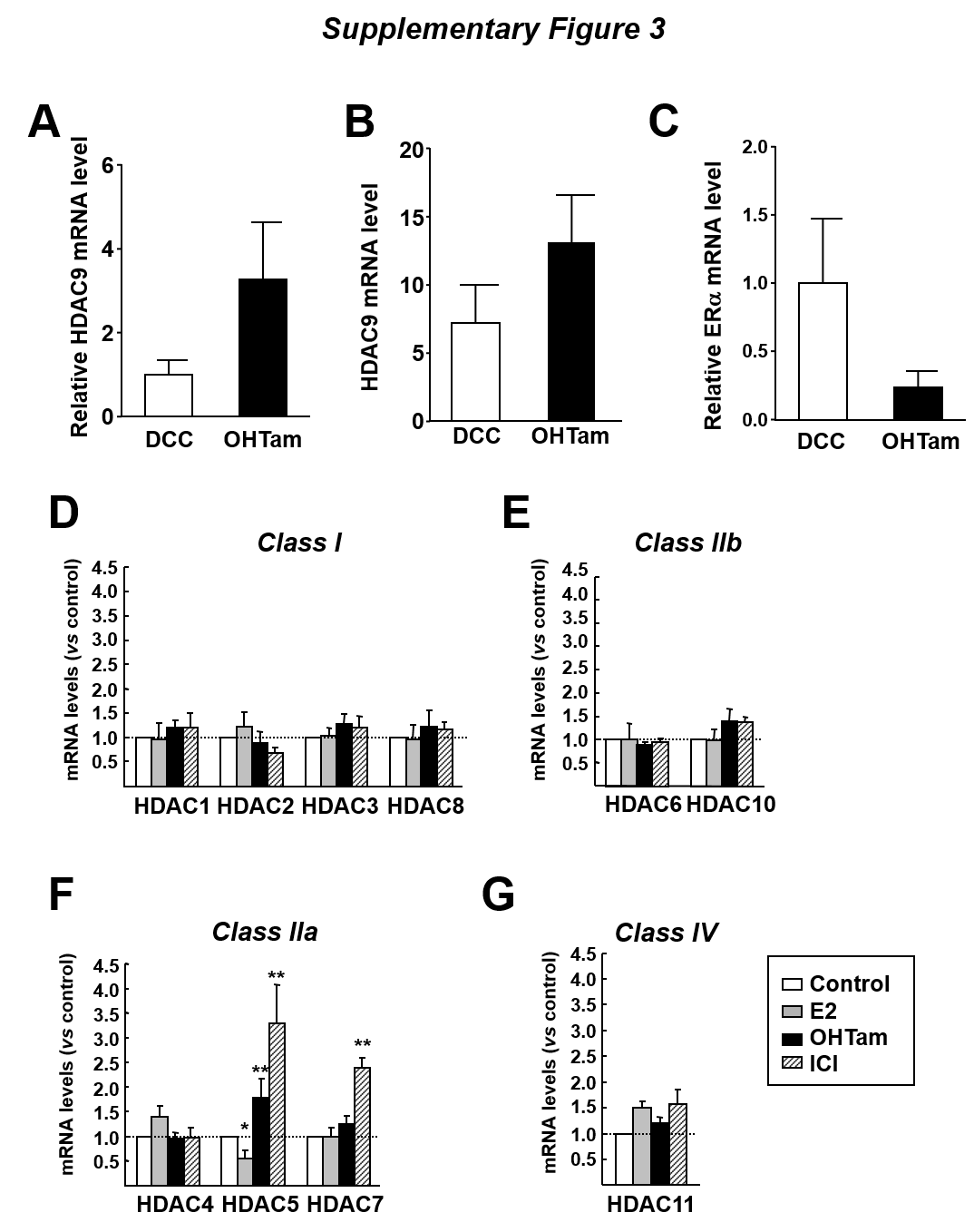
Supplementary Figure 3: Expression of HDAC9 mRNA in antiestrogen-resistant cells.***

**(A)** HDAC9 mRNA levels were quantified in antiestrogen-sensitive (DCC; controls) and -resistant (OHTam) MCF7 cells by RT-qPCR. Results are expressed relative to the HDAC mRNA levels measured in controls. Results are the mean ± SD of 3 independent cell cultures. **(B)** HDAC9 expression levels (205659_at probe set) were extracted from the GEO profile dataset GSE26459 and compared in tamoxifen-sensitive (DCC; controls) and -resistant (OHTam) MCF7 cells (18) (p<0.001; Mann-Whitney test). Statistical analysis was done with GraphPad Prism 5. **(C)** Same as in panel A for ERα mRNA levels. **(D-G)** MCF7 cells were incubated or not (control) with 10^-8^ M E2, OHTam, ICI or solvent alone (EtOH, Control) for 20h, and then class I (D), class IIa (E), class IIb (F) and class IV (G) HDAC mRNA levels were quantified by RT-QPCR. Results are expressed relative to the HDAC mRNA levels in control cells; *p<0.05, **p<0.01, Mann-Whitney test compared with control cells). Results are the mean ± SD of 3 independent cell cultures.


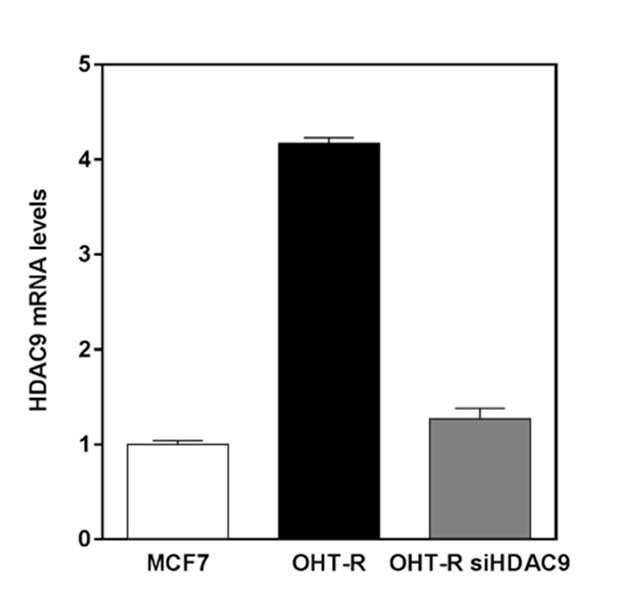


***Supplementary Figure 4: SiRNA-mediated knock-down of HDAC9 expression in***

***MCF7-OHTR cells.***

RT-qPCR analysis of HDAC9 expression in MCF7 and MCF7-OHTR cells transfected or not with a siRNA directed against HDAC9. The results are expressed in arbitrary units (AU) and represent fold change after normalization to 28S mRNA and to the level measured in MCF7 cells. Values are the means ± S.D.; n=3.


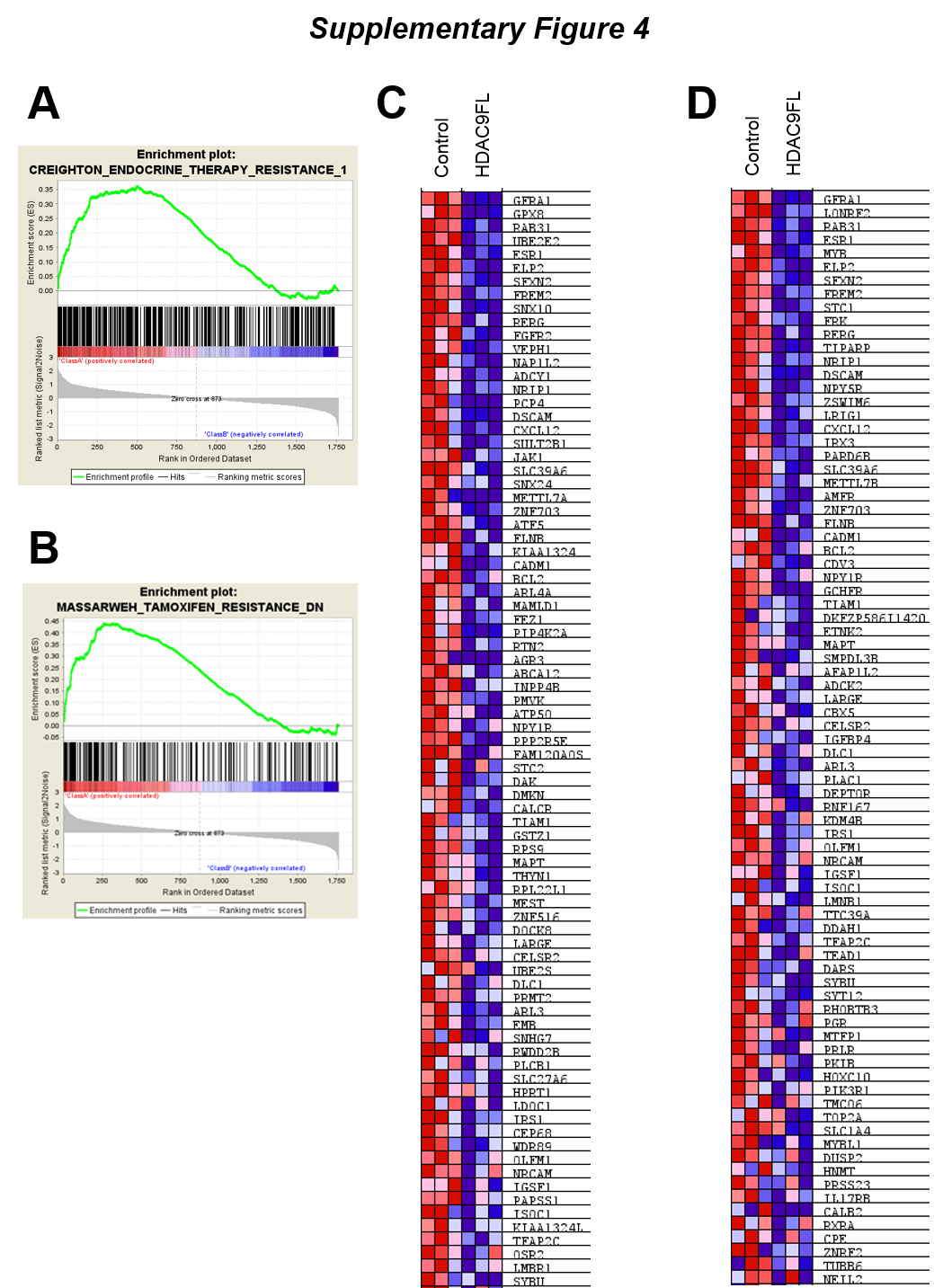


***Supplementary Figure 5: GSEA analysis of genes that are differentially regulated by HDAC9 in breast cancer cells.***

**(A-B)** GSEA results showing a signiﬁcant enrichment (p<0.05) of the “CREIGHTON ENDOCRINE THERAPY RESISTANCE 1” and “MASSARWEH TAMOXIFEN RESISTANCE DN” categories among the genes downregulated in the previously described MCF7-HDAC9FL *vs* MCF7-Control cells (8). **(C-D)** Heat maps show the expression level (red, up; blue, down) of the corresponding genes in the two cell lines.


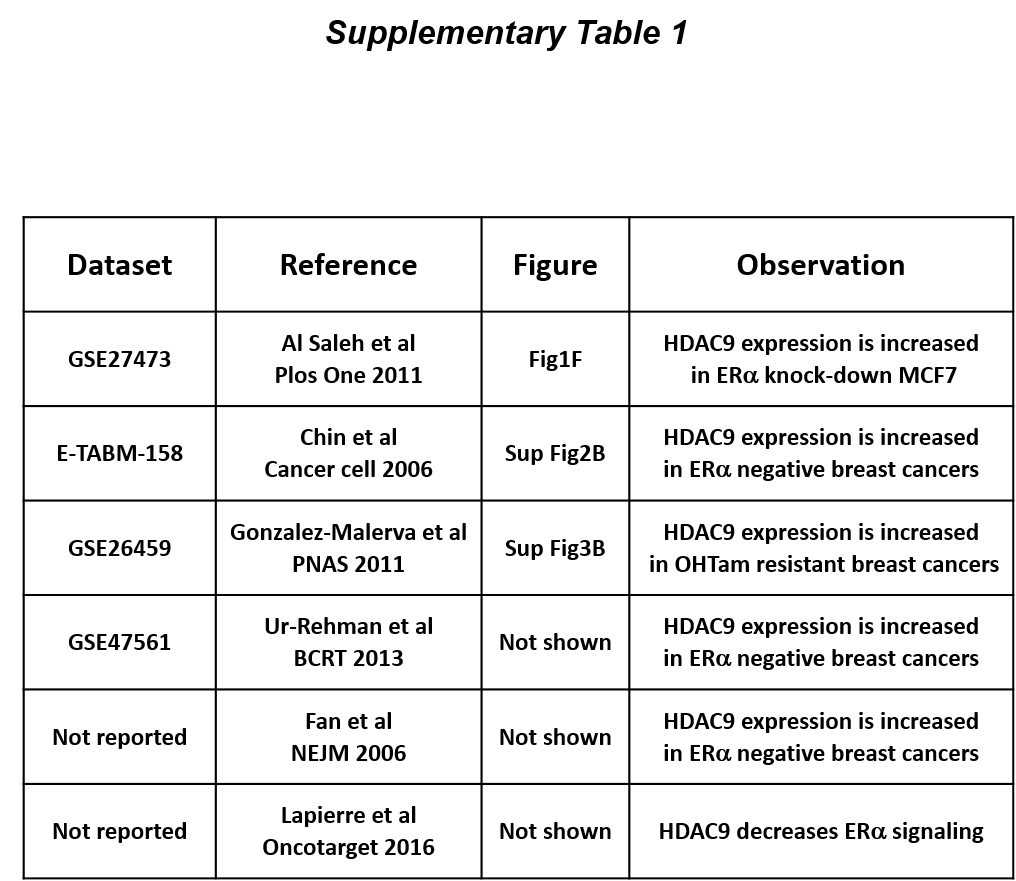


***Supplementary Table 1:*** List of the different datasets reanalyzed with the corresponding reference and the main observation obtained from their use.

***
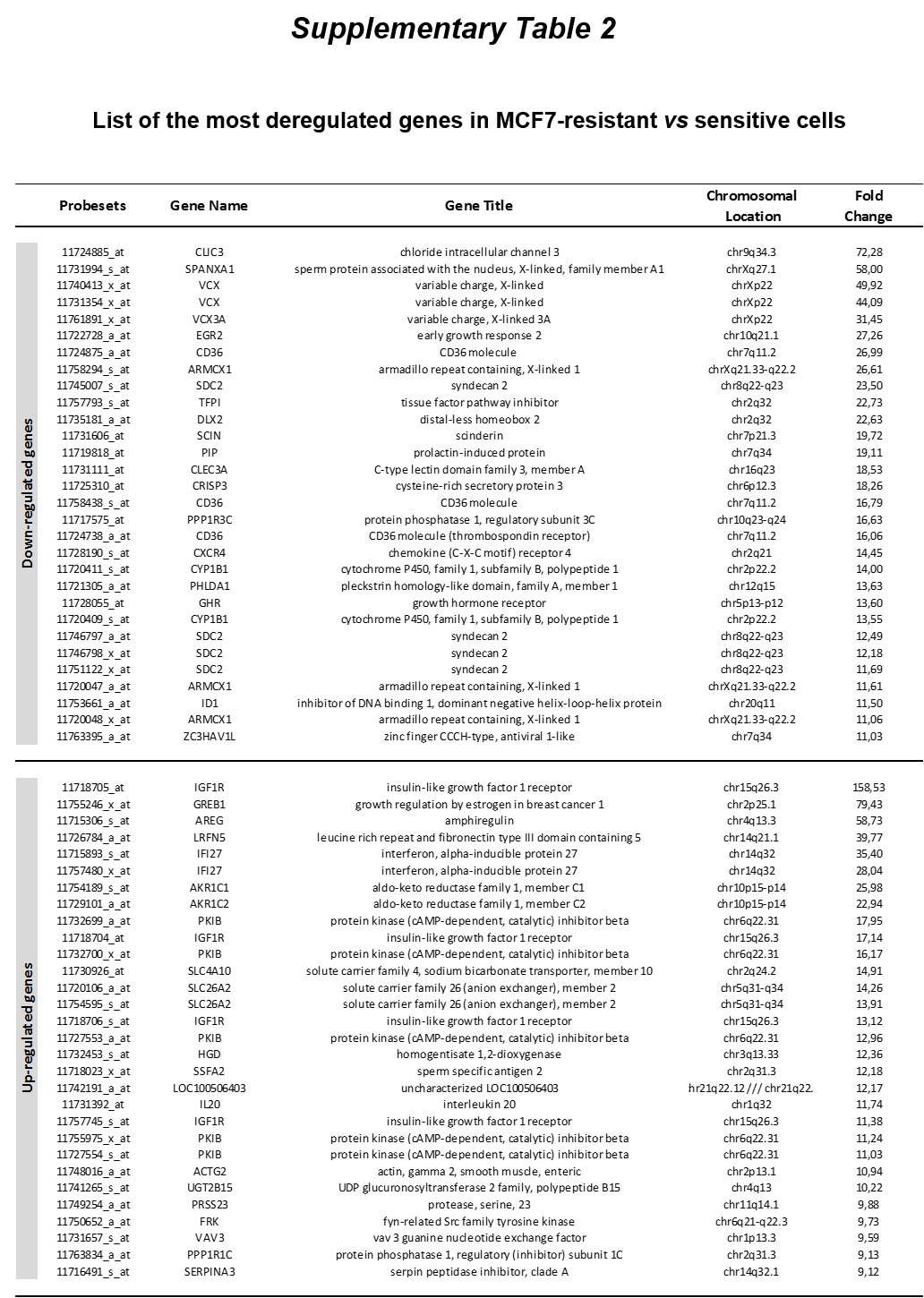
***

***Supplementary Table 2:*** List of the most deregulated genes in OHTam-resistant *vs* OHTam-sensitive MCF7 breast cancer cells.

***
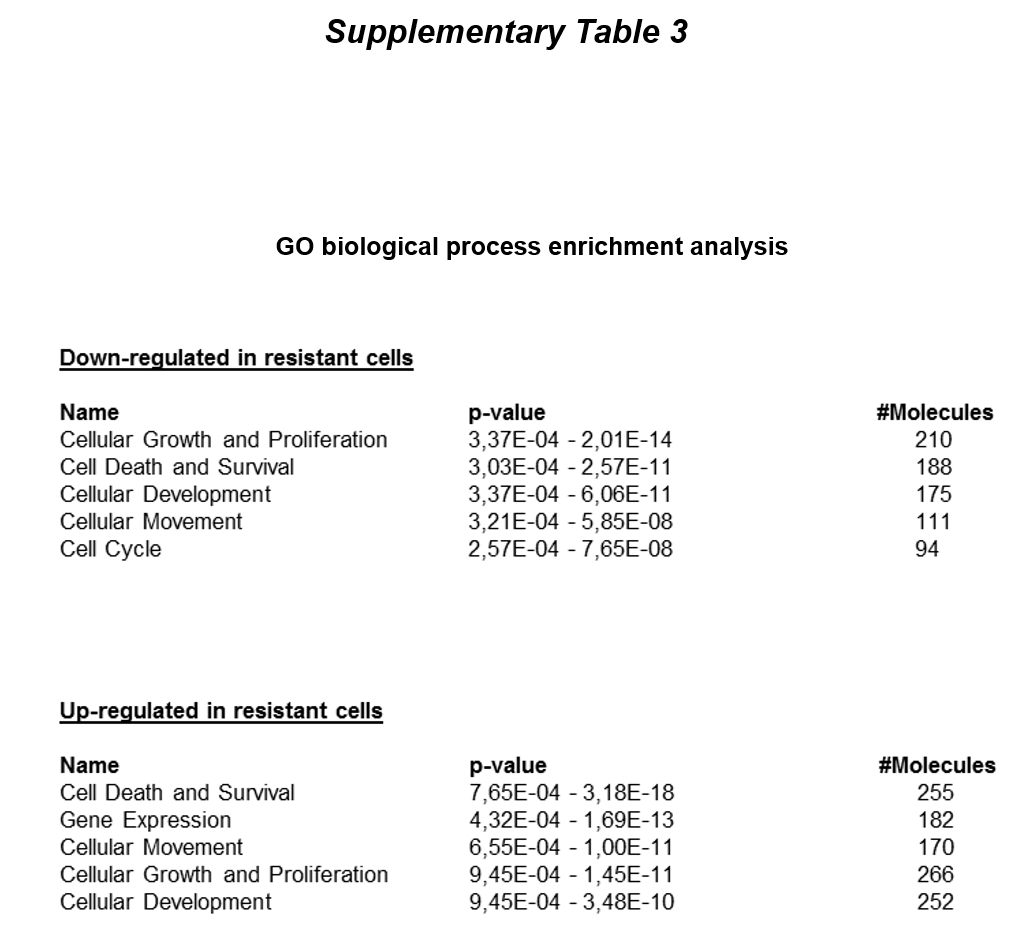
***

***Supplementary Table 3:*** List of the most deregulated GO biological profiles in OHTam-resistant *vs* OHTam-sensitive MCF7 breast cancer cells.

***
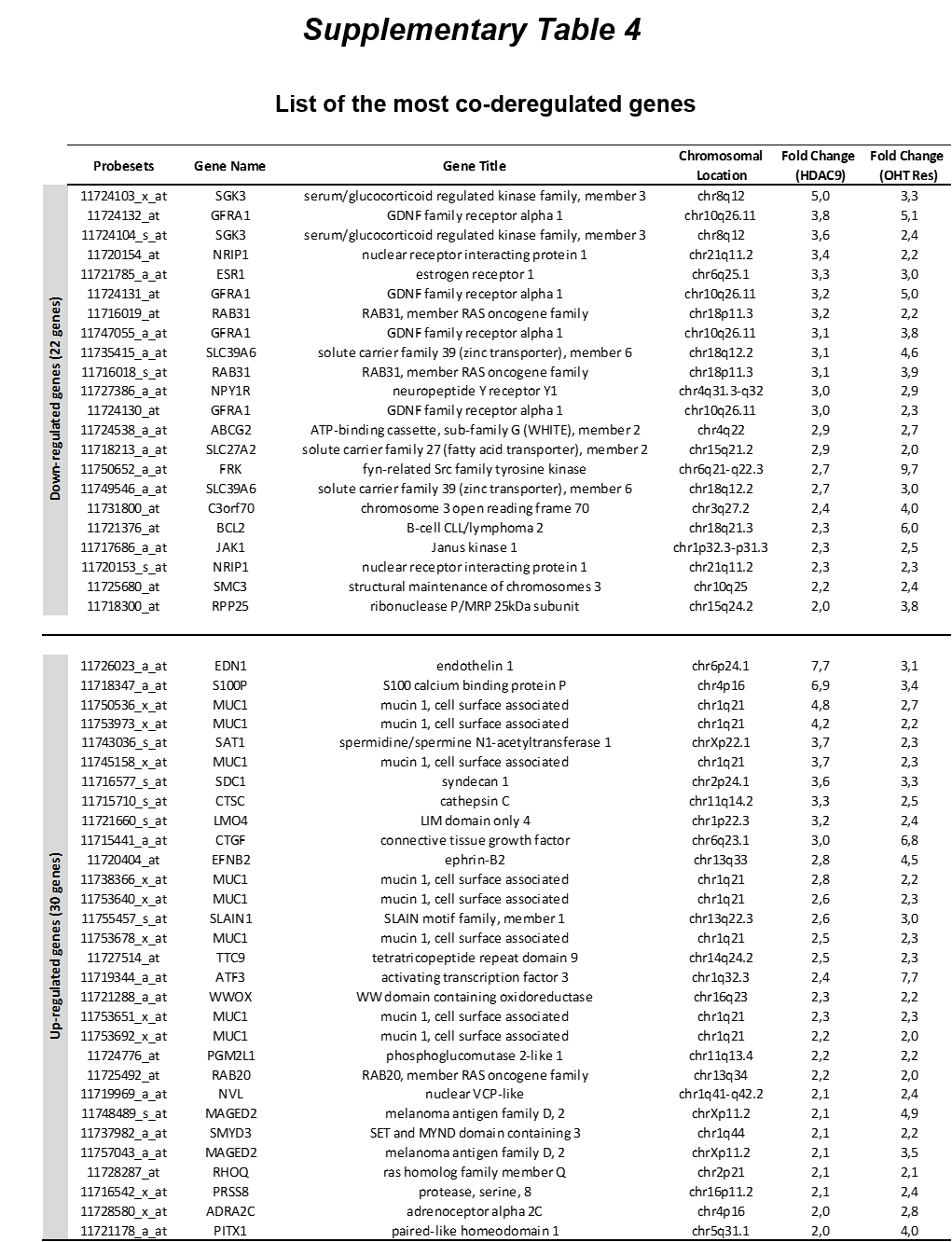
***

***Supplementary Table 4:*** List of the most common deregulated genes in OHTam-resistant and HDAC9-overexpressing MCF7 cells.
